# Genetic Diversity Assessment and Cultivar Identification of Cucumber (*Cucumis sativus* L.) Using the Fluidigm Single Nucleotide Polymorphism Assay

**DOI:** 10.3390/plants10020395

**Published:** 2021-02-19

**Authors:** Girim Park, Yunseo Choi, Jin-Kee Jung, Eun-Jo Shim, Min-young Kang, Sung-Chur Sim, Sang-Min Chung, Gung Pyo Lee, Younghoon Park

**Affiliations:** 1 Department of Horticulture Bioscience, Pusan National University, Miryang 50463, Korea; rlfla007@pusan.ac.kr (G.P.); chldudal1000@naver.com (Y.C.); 2 Seed Testing and Research Center, Korea Seed & Variety Service, Gimcheon 39660, Korea; jinkeejung@korea.kr (J.-K.J.); sej7742@korea.kr (E.-J.S.); kmyjj3802@korea.kr (M.-y.K.); 3 Department of Bioresources Engineering, Sejong University, Seoul 05006, Korea; sungchur@sejong.ac.kr; 4 Department of Life Sciences, Dongguk University, Seoul 04620, Korea; smchung@dongguk.edu; 5 Department of Plant Science and Technology, Chung-Ang University, Ansung 17546, Korea; gplee@cau.ac.kr

**Keywords:** cultivar group, germplasm assessment, intellectual right protection, molecular marker, population genetics

## Abstract

Genetic diversity analysis and cultivar identification were performed using a core set of single nucleotide polymorphisms (SNPs) in cucumber (*Cucumis sativus* L.). For the genetic diversity study, 280 cucumber accessions collected from four continents (Asia, Europe, America, and Africa) by the National Agrobiodiversity Center of the Rural Development Administration in South Korea and 20 Korean commercial F1 hybrids were genotyped using 151 Fluidigm SNP assay sets. The heterozygosity of the SNP loci per accession ranged from 4.76 to 82.76%, with an average of 32.1%. Population genetics analysis was performed using population structure analysis and hierarchical clustering (HC), which indicated that these accessions were classified mainly into four subpopulations or clusters according to their geographical origins. The subpopulations for Asian and European accessions were clearly distinguished from each other (*F*_ST_ value = 0.47), while the subpopulations for Korean F1 hybrids and Asian accessions were closely related (*F*_ST_ = 0.34). The highest differentiation was observed between American and European accessions (*F*_ST_ = 0.41). Nei’s genetic distance among the 280 accessions was 0.414 on average. In addition, 95 commercial F1 hybrids of three cultivar groups (Baekdadagi-, Gasi-, and Nakhap-types) were genotyped using 82 Fluidigm SNP assay sets for cultivar identification. These 82 SNPs differentiated all cultivars, except seven. The heterozygosity of the SNP loci per cultivar ranged from 12.20 to 69.14%, with an average of 34.2%. Principal component analysis and HC demonstrated that most cultivars were clustered based on their cultivar groups. The Baekdadagi- and Gasi-types were clearly distinguished, while the Nakhap-type was closely related to the Baekdadagi-type. Our results obtained using core Fluidigm SNP assay sets provide useful information for germplasm assessment and cultivar identification, which are essential for breeding and intellectual right protection in cucumber.

## 1. Introduction

Cucumber (*Cucumis sativus* L.) is a major fruit vegetable belonging to the family Cucurbitaceae. It has two primary varieties: cultivated cucumber (*C. sativus* var *sativus*) and its wild relative (*C. sativus* var. *hardwickii*) [1]. Cucumber is a diploid (2n = 2x = 14) with a small genome size of approximately 367 Mbp [2], and it is recognized as a genetically useful model plant species in terms of a fast generation cycle [3] and diverse sex expression patterns [4]. Cucumbers are the most widely cultivated vegetables globally, after tomatoes, onions, and cabbages [5]. Worldwide, approximately 75 Mton of cucumbers are produced, of which 75% (56.2 Mton) is produced in China, followed by Iran, Turkey, and Russia [6]. In Korea, approximately 0.3 Mton of cucumbers was produced in 2018, making it the world’s 16th largest producer [6].

After early domestication in India, China, and Western Asia, cucumber was introduced to Europe and the Americas [7]. Because of climatic and cultivation conditions or dietary preference during domestication, modern cultivars have diversified into the following four major types: Huánan-type (southern China), Huábei-type (northern China), European-type, and a crossbreed of Huánan × Huábei. European-type cultivars are subdivided into the English forcing group, grown for greenhouse production; slice group, sold fresh for salads; and pickle group, primarily used for processing or pickling. Cucumbers cultivated in South Korea are classified into three cultivar groups (types): Baekdadagi-, Nakhap-, and Gasi-types. Based on the climatic adaptations and phenotypic characteristics, the Baekdadagi- and Nakhap-types can be categorized as Huábei-type, while the Gasi-type can be considered as Huánan-type. Despite the distinct morphological variation in cultivar types, limited genetic diversity exists in cucumber due to severe bottlenecks encountered during domestication [8,9]. Currently, the National Agrobiodiversity Center (NAC) of the Rural Development Administration (RDA), South Korea, has reported at least 656 cucumber accessions, including breeding lines and cultivars (http://genebank.rda.go.kr accessed on 12 May 2020). According to the records of the Korea Seed & Variety Service (KSVS), approximately 50 commercial F1 hybrids have been registered from 2005 to 2020, and 547 cultivars have been reported for production and sale (http://seed.go.kr accessed on 12 May 2020). Among them, the Baekdadagi-type is approximately 25%, accounting for the largest percentage of annual production [10].

Evaluation of genetic diversity is critical for the management, conservation, and breeding processes of genetic resources [11]. Furthermore, plant cultivar identification is essential for registering new cultivars and plant variety protection (PVP). Currently, cultivars are identified using distinctness, uniformity, and stability (DUS) testing [12], which is based on morphological characteristics. DUS testing has the following drawbacks: prone to inconsistency, limited informative value, time-consumption, and low precision, particularly in instances where traits are quantitative [13,14]. By contrast, molecular markers based on DNA sequence polymorphisms can be used to effectively evaluate the genetic characteristics and diversity of groups and differentiate cultivars such that the genotype can be accurately identified during the seedling stage without the effect of the cultivation environment [15]. In addition, if the molecular marker information of the cultivar is used in the new cultivar registration process, the duration, space, and cost of the existing DUS test can be markedly reduced [12,14].

The genetic diversity of cucumbers has been evaluated based on the following types of molecular markers: isozyme [16,17,18], restriction fragment length polymorphism (RFLP) [19], random amplified polymorphic DNA (RAPD) [20], amplified fragment length polymorphism (AFLP) [21], and simple sequence repeat (SSR) [22]. Among these, RAPD has limitations owing to low interlaboratory reproducibility [23], while RFLP and AFLP are expensive [11]. SSR has the advantage of being codominant and multiallelic, but it has limited application in an automated genotyping system [24]. In contrast, single nucleotide polymorphisms (SNPs) are codominant markers that appear evenly with the highest frequency in the genome [25,26]. Sufficient numbers of genome-wide SNPs can be obtained from Genotyping-by-sequencing (GBS), which is a next generation sequencing-based genotyping method using relatively inexpensive and straightforward protocols [27]. In particular, these SNPs can be utilized in an automated genotyping platform [23,28] using a wide range of technologies such as the Fluidigm SNP assay [29], high-resolution melting (HRM) [30], and kompetitive allele-specific polymerase chain reaction (KASP) [31]. The Fluidigm SNP assay uses a nanofluidic integrated fluidic circuit-based genotyping system. Compared with KASP and HRM, in the Fluidigm SNP assay the initial investment cost is high but the cost for genotyping each sample afterward is relatively low, and the analysis is easy to automate. In addition, the number of samples that can be analyzed simultaneously is small in comparison with that in a fixed array such as the Affymetrix Axiom chip, but the number of samples and SNPs can be controlled relatively flexibly [28]. 

Recently, a cucumber Fluidigm SNP assay was developed for 240 core SNPs (core SNP marker sets) selected from more than 10,000 SNPs discovered through GBS of 88 commercial F1 hybrids [32]. To date, however, the usability of this Fluidigm SNP assay for cucumber germplasm resources and F1 hybrids with diverse genetic backgrounds has not been reported. In the present study, we aimed to evaluate the genetic diversity of cucumber accessions deposited at the NAC of RDA, South Korea, by using the developed cucumber Fluidigm SNP assay and identifying the commercial F1 cultivars currently traded in Korea. To our knowledge, this is the first study to assess the genetic diversity of cucumber germplasm collection in South Korea using a large set of SNPs, and our results provide useful information for breeding and intellectual-right protection in cucumber.

## 2. Results

### 2.1. Fluidigm SNP Assay

**Figure 1:**
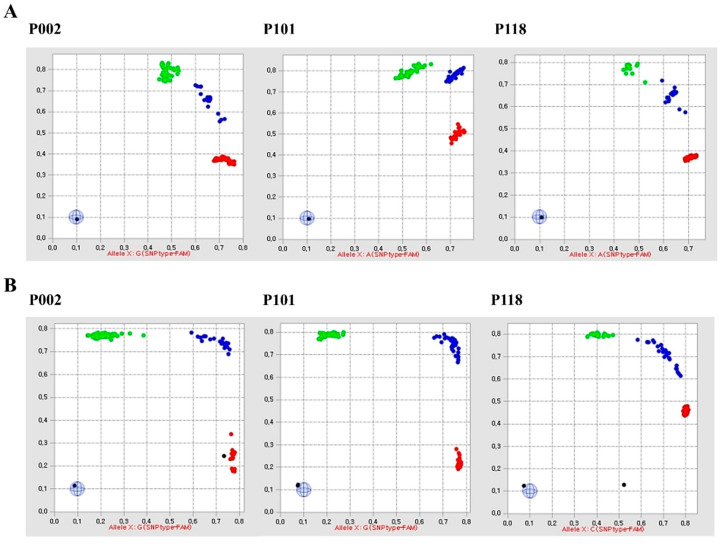
Scatter plots showing genotype calls for the single-nucleotide polymorphism (SNP) assay sets P002, P101, and P118 from the Fluidigm EP1^TM^ assay. Colored dots indicate SNP marker genotypes of the 280 cucumber accessions (**A**) and 95 commercial F1 hybrid cultivars (**B**). Red, blue, and green dots indicate XX (homozygote), YY (homozygote), and XY (heterozygote) genotypes, respectively. Gray and black dots indicate no call and no template control (NTC), respectively.

**Figure 2:**
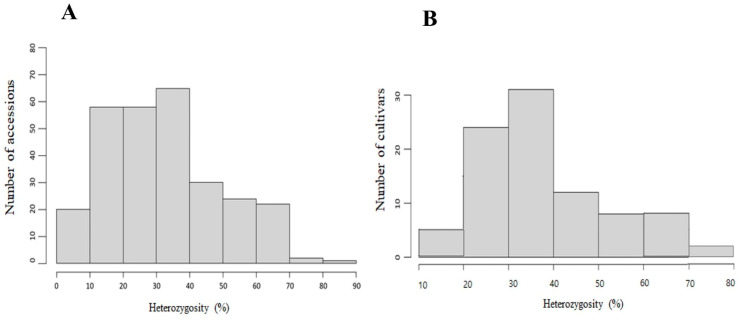
Distribution of the heterozygous marker genotype rate for the 280 accessions from the National Agrobiodiversity Center (NAC) (**A**) and 95 commercial F1 hybrids (**B**).

Genotyping results (Table S1) of 280 accessions using 151 Fluidigm SNP assay sets revealed that the success rate ranged from 17.88 to 100%, with an average of 95.2%. Scatter plots with genotype calls for three SNP assay sets (P002, P101, and P118) are shown in Figure 1A. The heterozygosity of the SNP loci per accession ranged from 4.76 to 82.76%, with an average of 32.1% (Figure 2A). The average heterozygosity of the SNP loci by continent was 36.72% in Europe (12 countries, 97 accessions), 31.05% in Asia (14 countries, 159 accessions), 20.93% in Africa (one country, one accession), and 18.07% in the Americas (three countries, 18 accessions). In terms of resource characteristics, 131 accessions were not disclosed (unknown accession; 30.53%), 63 accessions were marked as cultivars (37.12%), seven accessions were marked as breeding lines (9.07%), and 79 accessions were marked as heirloom, with an average heterozygosity of 32.80%. In addition, the average heterozygosity of the 20 PVP F1 hybrids was 29.80% (Table S1). The average heterozygosity of the SNP loci by cultivar group was 30.99%, 33.38%, and 27.42% for the Nakhap-, Gasi-, and Baekdadagi-types, respectively.

Genotyping of the 95 commercial F1 hybrids (Table S2) using 82 Fluidigm SNP assay sets showed a success rate of 95.12–100%, with an average of 99.02%. Scatter plots with genotype calls for three SNP assay sets (P002, P101, and P118) are shown in Figure 1B. The heterozygosity of the SNP loci per cultivar ranged from 14.80 to 74.47%, and the average was at 38.54% (Figure 2B). The average heterozygosity of the SNP loci by cultivar group was 45.41%, 43.80%, and 34.49% in Nakhap-, Gasi-, and Baekdadagi-types, respectively.

### 2.2. Population Genetics Analysis of 300 Accessions

#### 2.2.1. Population Structure

**Figure 3:**
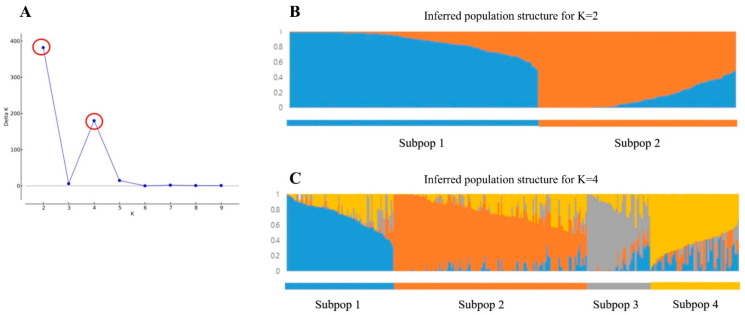
Population structure analysis of 300 cucumber accessions using 151 single-nucleotide polymorphism (SNP) assay sets. Population structure determined using an admixture-based clustering model. (**A**) Plot of delta K values with K ranging from 2 to 10 in the STRUCTURE analysis. (**B**) Population structure analysis results of cucumber accessions with K = 2 and (**C**) K = 4. Each accession is represented by a vertical bar. Each color represents one ancestral population, and the length of each colored segment of each vertical bar represents the proportion contributed by ancestral populations.

The population structure of 300 accessions was analyzed based on the genotyping results using 151 SNP markers. Estimation of the optimal number of subpopulations (subpops.) was found to be the most suitable when the subpopulations were classified into two (delta K = 2) and four (delta K = 4) (Figure 3A).

Population classification revealed that Subpop.1 was primarily composed of Asian accessions (ASs) and Korean PVP F1 hybrids (KF1s), while Subpop.2 mainly comprised European accessions (EUs), American accessions (AMs), and African accessions (AFs) (Table S3, Figure 3B). Within the four subpopulation classifications, Subpop.1 was chiefly composed of ASs and Gasi-type KF1s; Subpop.2, AMs and AFs; Subpop.3, Baekdadagi-type KF1s; and Subpop.4, EUs (Table S3, Figure 3C). Although most of the classifications were consistent with the continent-of-origin-based classification, high genetic admixture was observed in some accessions belonging to each subpopulation. In particular, the eight accessions of Uzbekistan, an Asian continental country, showed similar genetic composition to that of the AMs of Subpop.2. For the 20 PVP F1 hybrids, the Gasi- and Baekdadagi-types were classified into Subpop.1 and Subpop.3, respectively. Contrastingly, “Jewangcheongjang” and “Gyeoulsali” belonging to the Nakhap-type cultivars showed a genetic composition similar to that of the Baekdadagi-type. “Cheongjinju Nakhap”, “Mujinjang Nakhap”, and “Saeromi” cultivars showed a genetic admixture between Subpop.1 and Subpop.3 (Table S3, Figure 3). Among ASs, accessions originating from China, Japan, and Southeast Asian countries differed from each other. Similarly, Russian accessions among European countries were distinguished from those of other European countries. “Luckystrike” and “Asiastrike”, which are considered pickle-type cultivars based on their disclosed cultivar names, showed similar genetic composition in the STRUCTURE analysis results.

The expected heterozygosity (EH), representing the genetic diversity among individuals belonging to the subpopulations 1, 2, 3, and 4, were 0.3290, 0.2225, 0.3910, and 0.2206, respectively, while the observed heterozygosity (OH) was 0.2928, 0.2814, 0.2667, and 0.4839, respectively. The OH was higher than the EH for Subpop.2 and Subpop.4. The range of pairwise *F*st values calculated to estimate the genetic distance among the four subpopulations was 0.35–0.51, with an average of 0.43 (Table 1). The lowest *F*st value of 0.35 was between Subpop.1 and Subpop.3, and the highest *F*st value of 0.51 was between Subpop.2 and Subpop.4. Therefore, KF1s were genetically close to ASs and the farthest from EUs.

#### 2.2.2. Hierarchical Clustering (HC) Analysis

**Figure 4:**
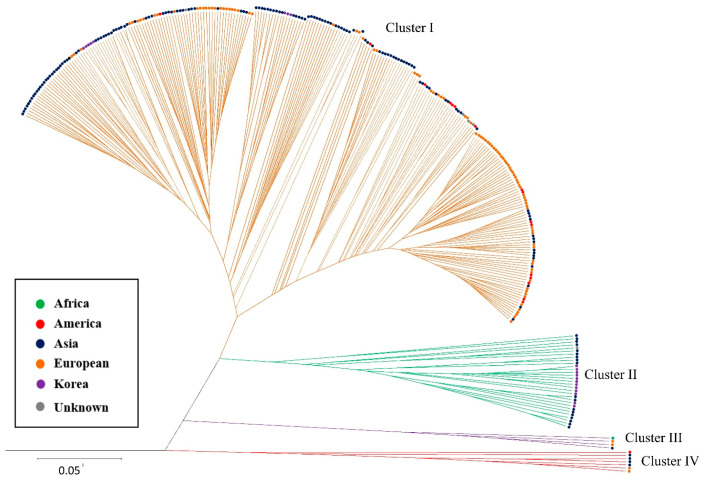
Unweighted pair-group method with arithmetic average (UPGMA) tree based on the genetic distance reported by Cavalli-Sforza and Edwards [32] for 300 cucumber accession using 151 SNP assay sets.

Through HC based on the genetic distance reported by Cavalli-Sforza and Edwards [33], it was possible to identify all 280 accessions and 20 KF1s, which were classified into four clusters (Figure 4). The cluster pattern was different from that of the four subpopulations in the STRUCTURE analysis; several subclusters were observed within Cluster I, consisting of the most accessions (n = 250) and eight KF1s (purple) excluding AFs. In particular, ASs (navy blue) and EUs (orange), which were classified into two subpopulations in the STRUCTURE analysis, showed independent subclusters in HC, and eight KF1s were located in the same subcluster as ASs. In Cluster II, 18 ASs were grouped with 13 KF1s, and in Cluster III, four accessions from AS, EU, and AM were grouped. In Cluster IV, seven EU, AM, and AF accessions were grouped together, and thus, the distinction between continents was not evident in minority accessions. 

### 2.3. Population Genetic Analysis of 95 Commercial F1 Hybrids

#### 2.3.1. Principal Component Analysis (PCA)

**Figure 5:**
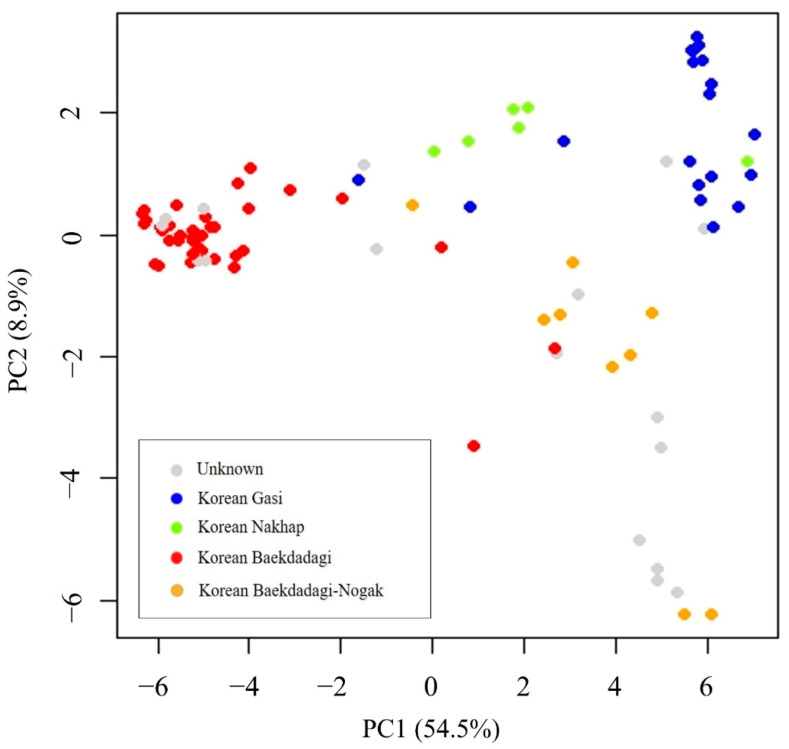
Principal component analysis (PCA) of the 95 commercial cucumber F1 hybrids using 82 single nucleotide polymorphism (SNP) assay sets.

PCA was performed based on the genotyping results of the 95 commercial F1 hybrids using 82 SNP assay sets. Consequently, three clusters were formed according to the cultivar group. Cluster I was primarily classified as Baekdadagi-type, Cluster II as Gasi-type, and Cluster III as Nakhap-type (Figure 5). However, the Baekdadagi-type cultivars indicated as “Nogak” (Korean Baekdadagi-Nogak) were not only classified in the Baekdadagi-type cluster but also showed differences from the other cultivar groups. Among the 17 F1 hybrids classified as “unknown”, seven were clustered with Baekdadagi-type, two with Gasi-type, and eight with Baekdadagi-Nogak. Based on PC1, the Nakhap-type cultivars tended to be closer to the Gasi-type than to the Baekdadagi-type cultivars.

#### 2.3.2. HC Analysis

**Figure 6:**
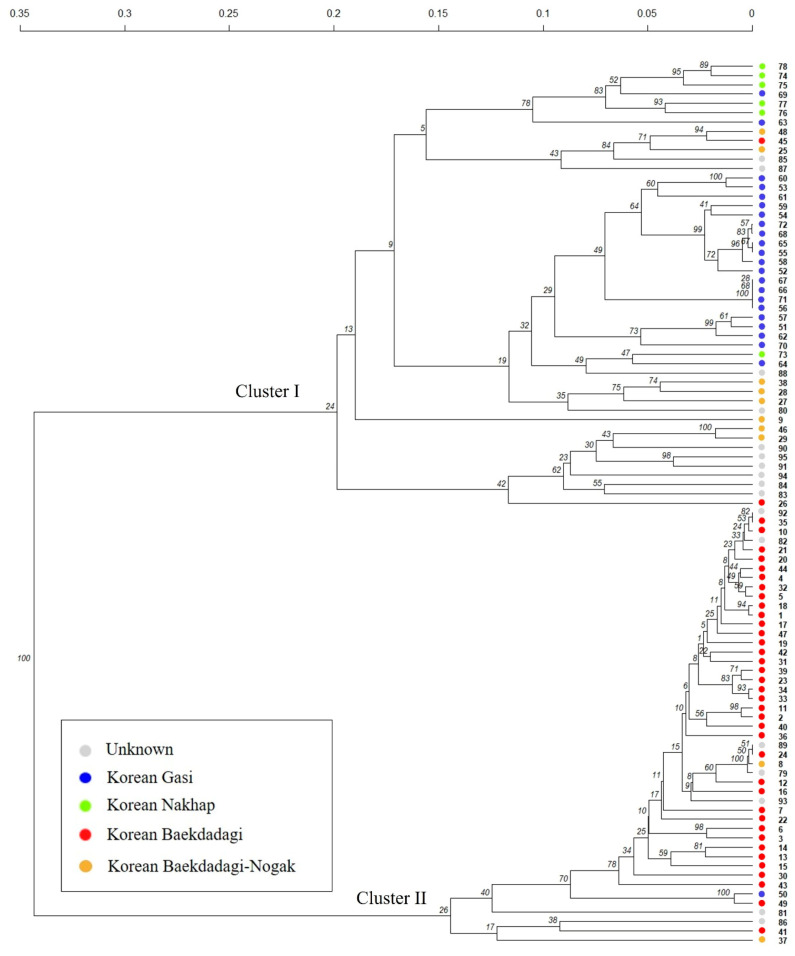
Hierarchical clustering based on Nei’s genetic distance of 95 commercial cucumber F_1_ hybrids using 82 single-nucleotide polymorphism (SNP) assay sets.

Based on the results of the analysis of 95 commercial F1 hybrids using 82 SNP assays, Nei’s genetic distance was calculated and HC was performed (Figure 6). The analysis showed that the 95 cultivars were primarily divided into two clusters (Cluster I: 48 cultivars; Cluster II: 47 cultivars). Cluster I was subdivided into subclusters similar to that in PCA. In Cluster I, subclusters were formed mainly by Nakhap-type, Gasi-type, and cultivars whose information was difficult to obtain (unknown). Cluster II was primarily composed of Baekdadagi-type cultivars. The Nogak cultivar was predominantly grouped in Cluster I, but two cultivars, “Daenong Nogak” and “Myeongga Nogak”, were clustered with the Baekdadagi-type cultivars in Cluster II. For a small number of cultivars, the cluster pattern was contrary to the cultivar information. “Shinasiaeuncheon” was classified as a Baekdadagi-type cultivar, but it was located close to the Nogak cultivar in Cluster I. Furthermore, “Jeil Jjang” was classified as a Baekdadagi-type cultivar, but it formed a subcluster with the “unknown” cultivars. In the evaluation of the cultivar identification ability of the 82 markers used, it was possible to distinguish between cultivars except for 18 cultivars: within the Baekdadagi-type cultivars, it was impossible to distinguish between some cultivars such as “Mannadadagi-1” and “Mannadadagi-2” and between “Achimhae” and “Haemaji” (genetic distance = 0). It was also challenging to distinguish “Yunina Samcheok”, “Saerounpaldo Samcheok”, “Gangpung Samcheok”, and “Saengnong Gasi”, which belonged to the Gasi-type cultivars.

## 3. Discussion

To date, genetic diversity evaluation and cultivar identification of cucumbers have been performed using various types of markers. In particular, the use of SSR markers for genetic resource evaluation and cultivar identification has been reported in several studies [7,11,22,30,34]. The genetic diversity of 29 cucumber accessions has been evaluated using genomic SSR and expressed sequence tag-SSR [35]. Furthermore, the population structure and genetic diversity have been analyzed for 3342 cucumber accessions collected from China, the Netherlands, and the United States by using 23 SSR markers [36]. Recently, an analysis of the genetic diversity of cucumber accessions using a large number of SNPs has also been reported [32,34], and population genetic analyses and genome-wide association studies have been performed using GBS of 1234 cucumber accessions at the U.S. Department of Agriculture (USDA) [37]. 

In the present study, 151 SNPs of 240 core Fluidigm SNP assay sets, first developed for cucumber cultivars, were used to evaluate the genetic diversity and cultivar identification ability of cucumber accessions at the NAC of RDA and commercial F1 hybrids in South Korea. In the 280 collected accessions and 95 commercial F1 cultivars, the success rates of marker analysis were high (95.20% and 99.02%, respectively), indicating that the core Fluidigm SNP assay sets can be used for genotyping cucumber germplasm with diverse genetic origins. For the collected accessions, the average proportion of the heterozygous marker genotype was high (32.1%), which was similar to that of 20 PVP F1 hybrids (29.80%) or 95 commercial F1 hybrids (38.54%). In contrast, the proportion of the heterozygous marker genotype was relatively low at 9.07% in seven breeding lines considered to have a high genetic fixation level. This implied that the collected accessions were mostly F1 cultivars or genetically heterogeneous heirlooms. 

Population structure analysis results revealed that accessions with similar geographic locations were classified as having similar population structures. Broadly, ASs, including KF1s and accessions derived from other continents (EUs, AMs, and AFs), showed a tendency to be distinguished from each other, and even within the ASs, KF1s were clearly distinguished. However, high levels of genetic admixture were observed for several accessions in the subpopulation, which were presumed to be due to hybridization and migration among the continents. The EH, a general statistic for evaluating genetic variation within a population [37], was higher in Asian and KF1s (0.3290) than in American (0.2225) and European (0.2206) populations and can be explained in relation to the origin of cucumbers. Cucumbers originated in India [1] and were domesticated in China and Western Asia and then introduced to other regions [7]. Korean and Japanese cucumber cultivars are believed to have been introduced from China, which is geographically close, and subsequently differentiated into cultivars and accessions with various traits according to the consumers’ preferences and cultivation environment. For American and European populations, most of the resource characteristics classification information is unknown, but it is presumed to be F1 hybrids or heirloom with close genetic relationship, as they show low EH. When the EH and OH in each subpopulation were compared, OH was higher than EH for Subpop.2 and Subpop.4. The OH is the actual frequency of heterozygous genotypes at a given locus within the population, while EH, a statistic commonly used for assessing genetic variation within the population, describes the expected proportion of heterozygous genotypes showing Hardy–Weinberg equilibrium [36]. If the OH is lower than expected, as observed in Subpop.1 and Subpop.3, there may be an effect of inbreeding, and the population could be composed of genetically diverse inbreds. By contrast, a higher OH than EH, as observed in Subpop.2 and Subpop.4, can be found in a population formed by the recent interbreeding of two isolated populations that were each homozygous for different alleles, which would be the case for F1 hybrids.

Additionally, in the pairwise *F*_ST_ analysis [38], which is an index that determines the diversity between populations, the value between Subpop.1 (ASs and KF1s) and Subpop.3 (KF1s) was the lowest, while it was the highest (0.5128) between Subpop.2 (AMs) and Subpop.4 (EUs). The *F*_ST_ values among Subpop.1, Subpop.2, and Subpop.4 were also relatively high (0.3905 and 0.4708, respectively). According to Wang et al. [37], 1234 cucumber accessions were divided into two clusters; ASs and AMs/EUs were separated, while AMs and EUs showed similar genetic composition compared with that of other regions. Consistent with previously reported results, our results showed high genetic diversity between Asian and American/European populations. However, the value between the European and American populations was also high. In the geographic distribution of EUs, 314 EUs and 97 North American accessions are distributed throughout Europe [37]. The EUs used in the current study were 97 accessions from 12 countries, of which 61 lines (~67%) originated from Russia. Furthermore, 38 of these accessions had similar accession names (“KS-”); thus, it is not easy to verify whether these accessions represent Europe. Moreover, this seemed to be the reason for the different patterns in previous studies. HC also showed geographic distance as an essential factor in determining genetic diversity. Korean, Chinese, and Japanese accessions have genetic characteristics similar to those of accessions from other Southeast Asian countries; this is also the case with accessions from European countries in geographic proximity, except for Russia.

In the current study, 82 Fluidigm SNP assay sets were used to show that identifying the 95 commercial F1 hybrids is possible at a high level. These SNP markers were able to classify the 95 commercial F1 cultivars by cultivar group, and cultivar-specific SNP marker sets could be generated for 77 cultivars for identification. Considering that the cultivars used in this study were commercial registration F1 hybrids (not PVP F1 hybrids), it could not be ruled out that the indistinguishable cultivars were the same but with different names. Because the genetic diversity of cucumbers is narrow [8,9] and phenotypic characteristics of cucumber cultivars grown in Korea are similar within the same group, additional SNP markers are required for more accurate cultivar identification. In particular, 10 Nogak cultivars (Korean Baekdadagi–Nogak, yellow dots in Figure 5; Figure 6) classified as a Baekdadagi-type group showed genetic differences from other cultivars in the Baekdadagi-type group and other groups. In terms of morphology, Nogak cultivars have short (10–15 cm) and thick fruit with yellow skin and seeded flesh, which are distinct from the fruit of the Gasi- and Baekdadagi-types. Unlike other Korean cucumber types, Nogak cultivars are generally used for oriental pickling. Our results suggest that Nogak cultivars could be classified as an independent Korean Nogak-type group.

It is vital to understand the genetic relationships among cucumber accession to use them as breeding materials. In addition, efficient cultivar identification technologies that can protect cultivars and breeders’ intellectual property rights that are essential for improving the breeding industry. To the best of our knowledge, the present study is the first to provide information for developing and protecting cucumber cultivars using Fluidigm SNP assay sets. However, because our results were interpreted based on the limited resource information obtained from the NAC, further analysis with phenotypic information may add more value to what we have currently established. Our results can be useful for understanding the genetic characteristics underlying cucumber germplasm in South Korea for identifying new cultivars.

## 4. Materials and Methods

### 4.1. Plant Material

Of the 300 cucumber accessions used to assess genetic diversity, 280 accessions were obtained from the NAC of RDA (Jeonju, Korea) (http://genebank.rda.go.kr accessed on 12 May 2020). From these, 275 accessions from 30 countries across four continents (Asia, America, Africa, and Europe) were collected, excluding five accessions whose origin was not disclosed. The resource characteristics included 78 heirlooms, 63 cultivars, 7 breeding lines, 1 genetic material, and 131 accessions without classification information (Table S3). In addition, 20 domestic F1 hybrids registered for plant variety protection (PVP F1 hybrids) used for comparative analysis with the NAC accessions were obtained from the KSVS. These 20 F1 hybrids consisted of 10 Baekdadagi-type, 5 Gasi-type, and 5 Nakhap-type cultivars.

Commercial registration F1 hybrids registered for domestic production and sale (commercial F1 hybrids) were selected from the cultivar inventory of the KSVS. A total of 95 commercial F1 hybrids were selected: 49 Baekdadagi-type, 23 Gasi-type, 6 Nakhap-type, and 17 cultivars that were difficult to classify (Table S4). 

The classification of origin and resource characteristics of the 280 accessions used in this study followed the information disclosed by the NAC, and the cultivar groups of the 115 commercial F1 hybrids were classified according to the published cultivar names and opinions of cucumber breeders (Haeoreum Seed, Pyeongtaek, Korea). 

### 4.2. DNA Extraction 

Genomic DNA extraction for the Fluidigm SNP assay was performed by collecting the first fully expanded leaf from three seedlings per cultivar and then using a GeneAll^Ⓡ^GeneEx^TM^Plant kit (GeneAll Biotechnology Co., Ltd., Seoul, Korea) according to the manufacturer’s instructions. The extracted DNA sample was used after quantitative and qualitative analysis using a spectrophotometer (Infinium F-200, Nanodrop, Illumina Inc., San Diego, CA, USA) and then diluted to a concentration of 20 ng·µL^–1^.

### 4.3. Fluidigm SNP Assay

For genotyping 300 accessions for genetic diversity analysis, 151 of the 240 patent-pending cucumber Fluidigm SNP assay sets (allele-specific primer (ASP1 and ASP2), locus-specific primer, and specific amplification primer) [32] were used (Table S5).

For genotyping 95 commercial registration F1 hybrids for cultivar identification, 82 of the 240 Fluidigm SNP assay sets described above were used (Table S6). A total of 67 SNP assay sets were common to both assays. Fluidigm assay was performed according to the manufacturer’s protocol using the genotyping platform Fluidigm EP1^TM^ Juno System (Fluidigm Corp, South San Francisco, CA, USA). The SNPs were then called using the Fluidigm SNP Genotyping Analysis software (version 4.5.1).

### 4.4. Population Genetics Analysis 

The population structure of 300 accessions was analyzed using an admixture-based model (Markov chain Monte Carlo (MCMC)-based Bayesian model) and considering independent allele frequencies n STRUCTURE 2.3.4 [38]. The range for K values was set from 1 to 10, and for each K value, the analysis was repeated 10 times by setting a 10,000 burn-in period and 100,000 MCMC repeats after the burn-in period. The optimal number of subpopulations was chosen based on the delta K value (∆K) calculated by the Evanno method [38] using STRUCTURE HARVESTER [39].

Genetic distance between individuals in each subpopulation (expected heterozygosity) and pairwise *F*_ST_ values between subpopulations was calculated using STRUCTURE 2.3.4. The genetic distance between accessions was calculated as described by Cavalli-Sforza and Edwards [33] using PowerMarker version 3.25 [40] and grouped using the unweighted pair-group method with arithmetic average. A dendrogram was created using MEGA 7.0 software [41].

PCA of the 95 commercial F1 hybrids was conducted using the pcaMethods package in R [42]. An HC tree was constructed based on Nei’s genetic distance using the Poppr package in R [43]. Bootstrap values for the tree were determined based on 1000 replications.

## Figures and Tables

**Table 1:** Pairwise *F*_ST_ value estimates between subpopulation pairs.

	Pop1	Pop2	Pop3	Pop4
Pop1	-	0.3905	0.3476	0.4708
Pop2	0.3905	-	0.3793	0.5128
Pop3	0.3476	0.3793	-	0.4957
Pop4	0.4708	0.5128	0.4957	-

## Data Availability

All relevant data are within this article and its Supplementary Data file.

## Supplementary Materials

The following are available online at https://www.mdpi.com/2223-7747/10/2/395/s1. Table S1. Single nucleotide polymorphism (SNP) genotyping data for 300 cucumber accessions. Table S2. Single nucleotide polymorphism (SNP) genotyping data for 95 commercial cucumber F1 hybrids. Table S3. List of the 300 cucumber accessions used for Fluidigm single nucleotide polymorphism (SNP) assay. Table S4. List of the 95 cucumber F1 hybrid cultivars used for Fluidigm single nucleotide polymorphism (SNP) assay. Table S5. Flanking sequences of 151 Fluidigm single nucleotide polymorphism (SNP) arrays. Table S6. Flanking sequences of 82 Fluidigm single nucleotide polymorphism (SNP) arrays.
